# DNA Methylation Analysis in the Intestinal Epithelium—Effect of Cell Separation on Gene Expression and Methylation Profile

**DOI:** 10.1371/journal.pone.0055636

**Published:** 2013-02-08

**Authors:** Andreas C. Jenke, Jan Postberg, Timothy Raine, Komal M. Nayak, Malte Molitor, Stefan Wirth, Arthur Kaser, Miles Parkes, Robert B. Heuschkel, Valerie Orth, Matthias Zilbauer

**Affiliations:** 1 Department of Paediatric Gastroenterology, Hepatology and Nutrition, Addenbrooke’s Hospital, University of Cambridge, Cambridge, United Kingdom; 2 Department of Neonatology, HELIOS Children’s Hospital, Witten/Herdecke University, Wuppertal, Germany; 3 Division of Gastroenterology and Hepatology, Department of Medicine, Addenbrooke’s Hospital, University of Cambridge, Cambridge, United Kingdom; University of Colorado Denver, United States of America

## Abstract

**Background:**

Epigenetic signatures are highly cell type specific. Separation of distinct cell populations is therefore desirable for all epigenetic studies. However, to date little information is available on whether separation protocols might influence epigenetic and/or gene expression signatures and hence might be less beneficial. We investigated the influence of two frequently used protocols to isolate intestinal epithelium cells (IECs) from 6 healthy individuals.

**Materials and Methods:**

Epithelial cells were isolated from small bowel (i.e. terminal ileum) biopsies using EDTA/DTT and enzymatic release followed by magnetic bead sorting via EPCAM labeled microbeads. Effects on gene/mRNA expression were analyzed using a real time PCR based expression array. DNA methylation was assessed by pyrosequencing of bisulfite converted DNA and methylated DNA immunoprecipitation (MeDIP).

**Results:**

While cell purity was >95% using both cell separation approaches, gene expression analysis revealed significantly higher mRNA levels of several inflammatory genes in EDTA/DTT when compared to enzymatically released cells. In contrast, DNA methylation of selected genes was less variable and only revealed subtle differences. Comparison of DNA methylation of the epithelial cell marker EPCAM in unseparated whole biopsy samples with separated epithelium (i.e. EPCAM positive and negative fraction) demonstrated significant differences in DNA methylation between all three tissue fractions indicating cell type specific methylation patterns can be masked in unseparated tissue samples.

**Conclusions:**

Taken together, our data highlight the importance of considering the potential effect of cell separation on gene expression as well as DNA methylation signatures. The decision to separate tissue samples will therefore depend on study design and specific separation protocols.

## Introduction

Recent evidence has emphasized that epigenetic signatures such as DNA methylation are highly cell type specific [1]. For example, elegant studies on the haematopoetic system have demonstrated how even closely related cell types display distinct methylation profiles [2], [3]. Hence, cell purification should always be considered prior to the investigation of epigenetic mechanisms in mixed cell- or tissue-samples. This is of particular importance when it comes to investigating the potential role and regulation of epigenetic mechanism(s) in gastrointestinal (GI) health and disease which largely relies on analysis of mucosal tissue samples (i.e. biopsies and resection material) containing different cell types of variable composition. However, depending on protocols and reagents used, cell purification procedures may cause significant changes in gene expression as well as DNA methylation and hence represent a major confounding factor. Surprisingly, limited information is currently available on changes of gene expression or DNA methylation caused by cell separation procedures. Whereas isolation and purification in blood samples (e.g. isolation of peripheral blood mononuclear cells) is relatively straight forward, this is not the case for mixed tissue samples such as intestinal biopsies where intestinal epithelial cells (IEC) are most commonly released using reducing and chelating [4], [5] agents and/or enzymatic digestion [6], [7]. Such reagents and the associated conditions have the potential to cause stress-induced changes of gene expression and DNA methylation signatures.

The aim of this study was to explore the influence of different cell isolation methods on gene expression and DNA methylation profiles in IEC. Results of our study demonstrate major differences to changes in gene expression and to a much lesser degree in DNA methylation according to the separation method employed. This data further highlights the need to consider these potential effects prior to deciding if cell separation is required as well as at the stage of interpreting results obtained from pre-treated tissue samples.

## Results

Biopsies specimen were collected from the terminal ileum of six healthy individuals (4 male, 2 female). Median age was 60.5 years (range 41–83y). All patients underwent colonoscopy for colorectal cancer screening and were found to have macroscopically normal colons. All patients were non-smokers.

### Effectiveness of Isolation Procedures

Methods to purify IEC from mucosal biopsies rely on initial disruption of the epithelium. This can be accomplished chemically, through treatment with the chelating agent ethylenediaminetetraacetic acid (EDTA) and/or the reducing agent dithiothreitol (DTT) [4], [5]. Alternative approaches use various digestive enzymes to disrupt the basement membrane and extracellular matrix of the lamina propria, sometimes after an initial EDTA/DTT incubation. Subsequent purification of IEC from accompanying mononuclear and stromal cells may be accomplished by density centrifugation or cell sorting using immunomagnetic beads or flow cytometry. In order to study the effects of reagents used for the initial incubation, we obtained eight biopsies taken from the terminal ileum of individual patients. Four pooled biopsy samples were then subjected to either chemical digestion using a combination of EDTA and DTT, or enzymatic digestion with Liberase (a blend of collagenase I/II and a neutral protease; Roche Applied Science) and hyaluronidase. Both protocols were performed in parallel with similar overall processing times. Resulting cell suspensions were then used for immunomagnetic bead based positive selection of IEC using antibodies against the epithelial cell specific adhesion molecule CD326.

**Figure 1:**
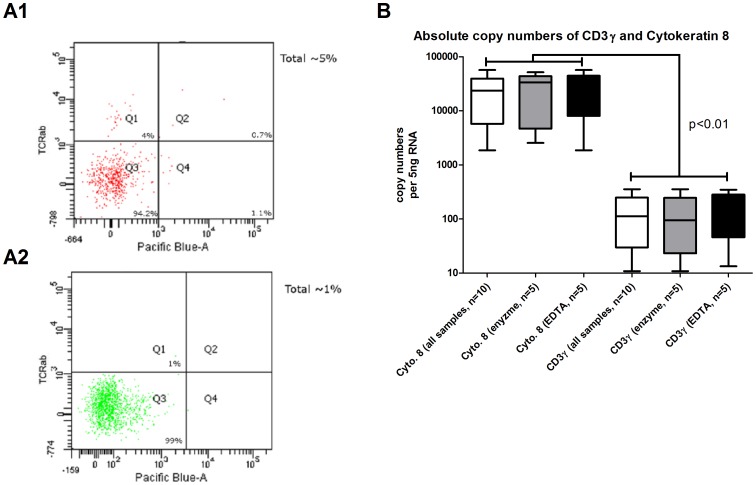
Cytokeratin 8 and CD3γ copy numbers in intestinal epithelial cells. A) FACS analysis of T cell contamination. Positive selection of IEC was performed using CD326 Microbeads followed by staining using T-cell receptor antibodies and Pacific Blue Annexin V to detect apoptotic cells. Panel A1 shows an example of IEC isolated using the enzyme based protocol, panel A2 using the EDTA/DTT based protocol. T-cell receptor positive cells are located in quadrant Q1 and Q2. Analysis of absolute mRNA copy numbers of cytokeratin 8 and CD3γ in intestinal epithelial cells separated by positive selection using CD326 Microbeads cells. B) Expression of mRNA copy numbers of Cytokeratin 8 and CD3γ. Data is expressed as box and whisker plots. The length of the boxes represents the interquartile range and the whiskers the 3^rd^ and 97^th^ percentile of the data. Significance testing was performed using paired Students T-test and values for *p*<0.05 were considered to be statistically significant.

First we aimed to compare the purification efficiency of the two separation protocols. Therefore purified cell samples were tested by flow cytometry for the presence of TCRαβ^+^ T cells, which represent the major contaminating cell type. T-cell contamination was found to be below 5% without significant differences between EDTA/DTT and enzymatically released cells whereas the yield of viable cells was slightly but not significantly higher in the EDTA/DTT based protocol (Fig. 1a). No significant differences in cell viability between both protocols were observed. Since surface markers might be cleaved during the extraction process, we also assessed T-cell contamination using quantitative PCR with primers for cytokeratin-8 and CD3γ. Copy numbers for cytokeratin were more than 200-fold higher than for CD3γ, with no significant differences between EDTA/DTT and enzymatically released cells (Fig. 1c).

### Effects of Dissociation Protocol on Gene Expression

**Figure 2:**
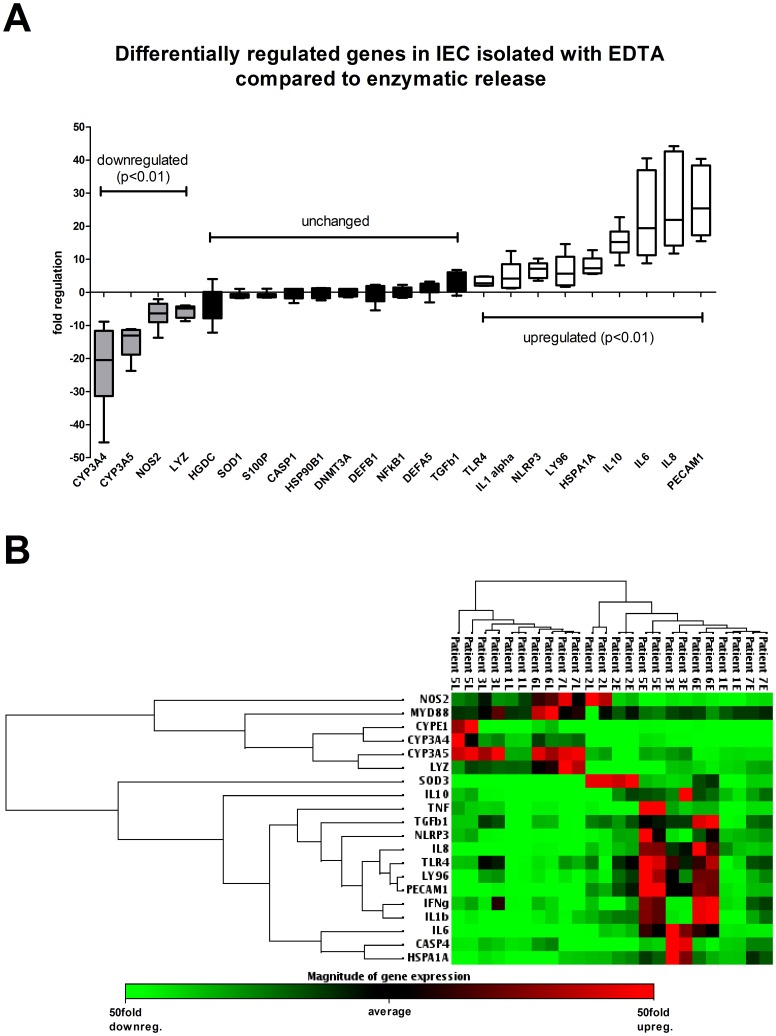
Influence of isolation methods on gene expression profiles. A) Differentially regulated genes in IEC isolated with EDTA/DTT compared to enzymatic release. Data is expressed as box and whisker plots. The length of the boxes represents the interquartile range and the whiskers the 3^rd^ and 97^th^ percentile of the data. Significance testing was performed using paired Students T-test and values for *p*<0.05 were considered to be statistically significant. B) Cluster analysis of all analyzed patients. Individuals are numbered. An L signifies IEC isolated using enzymatic release. An E signifies IEC isolated using the EDTA/DTT-based approach. All six patients were included in this analysis. Tissue samples for EDTA/DTT and enzymatic release were obtained from the same side in each patient and all analyses were performed in triplicate.

Removal of epithelial cells from the basement membrane is inherently stressful and associated with high rates of cell death [7]. To determine to what extent the methods used in the initial isolation might cause additional cellular stress, we used real time PCR based arrays to analyse 44 selected genes, including antimicrobial peptides (AMPs), inflammatory cytokines, pattern recognition receptors (PRRs) and chaperones. Copy numbers of mRNA of several inflammatory cytokines and PRRs was significantly higher in cells isolated using EDTA/DTT when compared to enzymatic release (Fig. 2a). For the used housekeeping genes ACTB, GAPDH, RPL13A and VIL1 as well as for genes not shown in figure 2a (PPC, CYPE1, IL4, RTC, S100A12, ATG16L1, SOD2, HSPD1, HSPE1, HSP90AA1, HSPA5, DDIT3, DEFA6, TLR2, DEFBA4, MYD88, CASP4, IFNγ, IL1b, TNF and SOD3) no significant differences were noted.

For example, mRNA copy numbers of PECAM1 were increased 27-fold, IL10 15-fold, HSPA1A 8-fold and TLR4 3-fold. In contrast, mRNA copy numbers of genes involved in regular cell function and maintenance were found to be significantly reduced in EDTA/DTT treated samples, with CYP3A4 (22-fold) and NOS2 (7-fold) being the most pronounced examples (Fig. 2a). Interestingly, there was no difference in mRNA copy numbers for the DNA methyltransferase DNMT3A, which is involved in epigenetic regulation by DNA de novo methylation. Hierarchical clustering analysis using the 20 most differentially expressed genes generated two pronounced gene clusters in which gene expression of maintenance and inflammatory genes were strongly correlated distinctly separating EDTA/DTT and enzymatically released cells (Fig. 2b).

### DNA Methylation Analysis of Selected Genes

**Figure 3:**
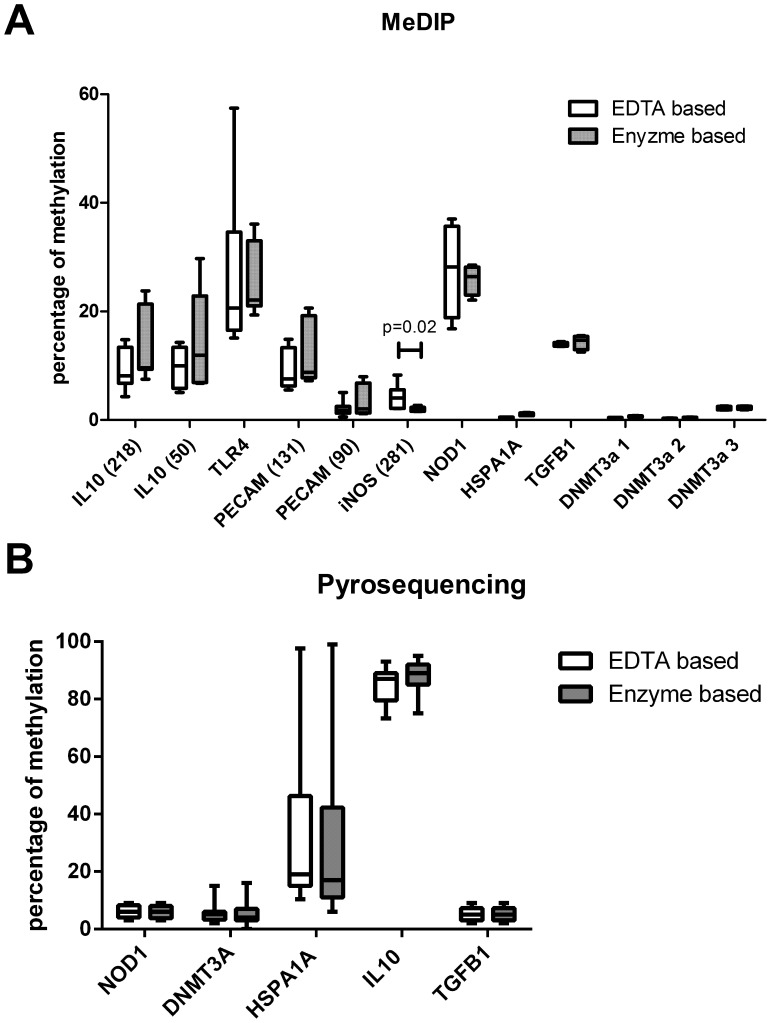
Analysis of cytosine methylation of promoter regions of selected genes. A) CpG methylation of promoter regions of selected genes analysed by MeDIP. B) CpG methylation of promoter regions of selected genes analysed by pyrosequencing. Data is expressed as box and whisker plots. The length of the boxes represents the interquartile range and the whiskers the 3^rd^ and 97^th^ percentile of the data. Significance testing was performed using paired Students T-test and values for *p*<0.05 were considered to be statistically significant.

We next selected differentially and non-differentially regulated genes for analysis of cytosine methylation of the corresponding promoter regions by methylated DNA immunoprecipitation (MeDIP). The selection was based on two criteria – the observed extent of differences in gene expression depending on cell isolation (Fig. 2) and the availability of published data demonstrating gene expression to be regulated by cytosine methylation. Differences in cytosine methylation between EDTA/DTT and enzymatically released cells were generally either completely absent or small (median 3.3%, range 2.4 to 5.0%) without reaching statistical significance. One exception was NOS2 (iNOS), which was significantly stronger methylated in EDTA/DTT when compared to enzymatically released cells (4.33% versus 1.97%, *p* = 0.02) (Fig. 3a). To confirm and further extend our data obtained using MeDIP, individual CpG methylation status of promoter regions of a subset of these genes were analyzed by pyrosequencing of bisulfite converted DNA. Importantly, pyrosequencing revealed similar results with no significant differences in DNA methylation between EDTA/DTT and enzymatically released cells the tested CpGs (Fig. 3b).

### Differences in DNA Methylation of the Epithelial Cell Marker EPCAM in Whole Biopsy Versus Separated Cell Fractions

**Figure 4:**
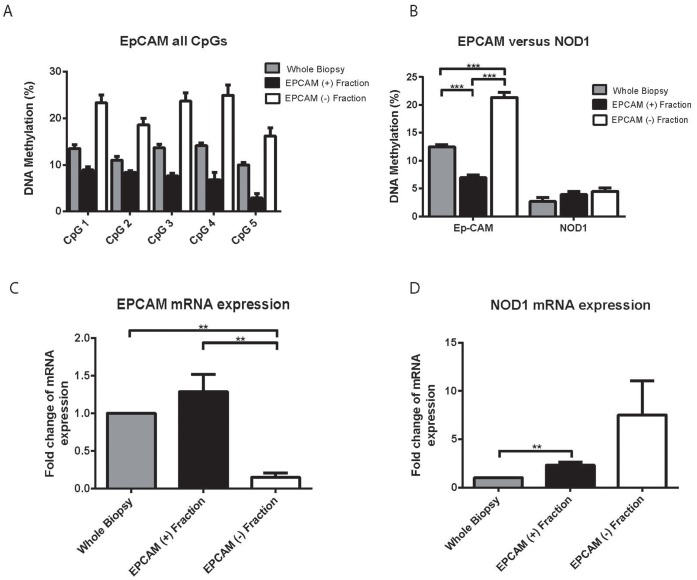
DNA methylation of EPCAM in whole biopsy samples compared to separated epithelium and negative cell fraction. A) DNA methylation of 5 CpGs within the EPCAM gene using a pyrosequencing assay. B) Total CpG methylation comparing EPCAM versus NOD1. Assays included 5 CpG sites for EPCAM and 2 for NOD1. C) EPCAM mRNA expression in whole biopsies compared to epithelial positive and negative separated cell fraction. Data is expressed as mean +/− SEM of 5 tissue samples. Differences were considered as statistically significant if p<0.05 (** p<0.001, ***p<0.0001).

Given the significant changes in mRNA expression induced by cell separation we next aimed to illustrate cell type specific DNA methylation in separated versus unseparated intestinal tissue. We therefore used DNA isolated from whole, unseparated biopsy material (i.e. terminal ileum) as well as the separated epithelial cell fraction obtained by enzymatic release and EPCAM labeled bead sorting. Additionally, we included the EPCAM negative (i.e. non epithelial) cell fraction, which will mainly consist of intraepithelial lymphocytes. We first assessed DNA methylation of 5 CpGs using pyrosequencing of bisulfite converted DNA within the EPCAM gene (epithelial cell marker). As demonstrated in Figure 4a, we observed consistent differences in DNA methylation of all 5 CpG sites with up to 20% lower levels of methylation in the EPCAM positive compared to EPCAM negative cell fraction. Methylation levels of whole biopsies were placed between both separated cell fractions. Differences in the overall methylation profile of all 5 CpGs were found to be highly statistically significant (Figure 4b). As expected, differences in EPCAM mRNA expression reflected the successful intestinal epithelial cell separation with significantly higher expression levels in the epithelial cell fraction (Figure 4c). In contrast to the epithelium specific gene EPCAM, no differences in DNA methylation between the whole biopsy, EPCAM positive and negative fraction were observed in a NOD1 pyrosequencing assay interrogating 3 CpG sites (Figure 4b).

## Discussion

The intestinal epithelium represents a crucial barrier in the GI tract and its malfunction has been implicated in several GI inflammatory and malignant conditions [8]. Moreover, epigenetic changes have been shown to occur in response to food substances and/or the bacterial microflora [9], [10]. It is therefore not surprising that several studies have demonstrated changes of epigenetic signatures such as DNA methylation in the GI mucosa of patients with inflammatory and/or malignant GI conditions [11], [12]. However, given the highly cell type specific character of epigenetic signatures, there is a risk that observed changes might simply reflect altered cell composition of mixed cell tissue samples such as influx of inflammatory cells during intestinal inflammation. Hence, isolation and purification of individual cell subsets such as the intestinal epithelium should be considered in order to pick up cell type specific epigenetic changes such as DNA methylation profiles. Unfortunately, isolating IEC from mucosal biopsies is inherently stressful for the cells, which may risk confounding results by causing changes to gene expression and DNA methylation.

In our study we investigated the impact on gene expression and DNA methylation of two frequently used sets of reagents in the isolation of IEC from mucosal biopsies, i.e. EDTA/DTT versus enzymatic release. Although previous reports have suggested that enzymatic digestion in the absence of EDTA may lead to incomplete release of epithelial cells from crypts [7], in our hands both methods performed equally well in achieving >95% purity of extracted IEC with minimal T-cell contamination. Importantly, we observed significantly higher mRNA expression levels of several inflammatory genes in EDTA/DTT treated IEC compared to those released by enzymatic digestion alone. Moreover, a number of maintenance function genes such as CYP3A and NOS2 showed significantly reduced mRNA levels in EDTA/DTT treated cells possibly reflecting a more toxic effect of these chelating agents [13]–[15]. In contrast to gene expression, DNA methylation did not seem to be effected as much as gene expression. Specifically when MeDIP was used for DNA methylation analyses, only methylation of the NOS2 promoter region was significantly stronger in EDTA/DTT treated cells. However, further analysis using pyrosequencing of bisulfite converted DNA did not reveal any differences in DNA methylation in the tested CpGs. Not surprisingly, mRNA copy numbers of DNMT3A did not differ in both methods. The reason for the observed difference in DNA methylation at the NOS2 promoter remains speculative. One may hypothesize that this finding reflects a subtle global change in DNA methylation and a genome wide DNA methylation analyses would be required to address this question. In the final part of our study we provide one example of cell type specific DNA methylation pattern that can only be observed in purified tissue samples. Again, future studies are required to demonstrate these differences on a whole genome level.

In summary, our data reveal substantial differences in the effect of cell separation protocols on gene expression whereas DNA methylation appears to remain more stable. We provide further evidence that cell separation is required to identify cell type specific methylation patterns. Importantly, both aspects have to be carefully considered by weighing up advantages and disadvantages of cell separation prior to starting DNA methylation analysis on mixed cell tissue samples such as intestinal biopsies.

## Materials and Methods

Eight pinch biopsies were taken from terminal ileum (TI) of healthy individuals and incubated for 10 min at RT in red blood cell lysis buffer (Roche Applied Science, Germany) to remove erythrocytes. Samples were then washed twice with HBSS medium. Written informed consent for research biopsies was obtained from all patients prior to endoscopy. Ethical approval for this study was obtained from the local NHS research ethics committee (East of England Cambridge and North West London).

### Enzymatic Cell Separation

Samples were incubated in HBSS medium containing 1.07 Wünsch units/ml Liberase DH (Roche Applied Science, Germany) and 70 U/ml hyaluronidase (Calbiochem/Merck, Germany) for 75 min at 37°C on a shaking platform at 600 rpm. Every 15 min the solution was homogenized by pipetting up and down for 1 min. Cells were then passed through a 40 µM sieve, spun down at 300 g at 4°C for 10 min and resuspended in 300 µl PBS buffer containing 100 IU/ml DNAse II (Sigma Aldrich, Missouri, USA).

### EDTA/DTT Based Cell Separation

Samples were incubated in HBSS medium containing 4 mM EDTA and 2 mM DL-Dithiothreitol (DTT, Sigma Aldrich) under vigorous shaking of 1750 rpm for 25 min at room temperature. 2 mM fresh DTT was added after 15 min. The cell suspension was then vortexed for 4 min. After leaving to settle, 13 ml of the supernatant was collected, stored on ice and replaced by 8 ml HBSS medium containing 0.5% BSA and 2 mM EDTA. The suspension was vortexed for a further 4 min and the supernatant was collected. The remaining 2 ml were vortexed again for 4 min and then passed through a 40 µM sieve. The flow through was then pooled with the previously collected supernatants. Cells were spun down at 300 g at 4°C for 10 min and resuspended in 300 µl PBS buffer containing 100 IU/ml DNAse II and 2.5 mM EDTA.

### Positive Selection for Epithelial Cells and FACS Analysis

Positive selection for epithelial cells was performed by magnetic separation with human CD326 Microbeads (Miltenyi, Bergisch-Gladbach, Germany) following manufacturers’ instructions with minor modifications. In particular, magnets, columns and buffers were kept at 4°C before and during use, and the MACS buffer was supplemented with 100 IU/ml DNAse II (Sigma Aldrich). For determination of T cell contamination 1×10^5^ cells were surface stained with PE/Cy5 anti-human TCRα/β (Biolegend, New Jersey, USA) for 30 min at room temperature. For detection of apoptotic cells Pacific Blue-Annexin V staining was used (Biolegend, New Jersey, USA).Cells were then spun down for 15 min at 40 g and then resuspended in PBS. FACS analysis was performed on a ImageStream (Amnis, Seattle, USA). T cell contamination was less than 5% in all samples.

### RNA/DNA Extraction and Reverse Transcription PCR

After standard RNA extraction using the AllPrep DNA/RNA kit (Qiagen, Hilden, Germany) integrity of extracted RNA was analyzed by agarose gel electrophoresis and quantified using spectrophotometric absorbance at 260 nm. Thereafter 500 ng of RNA were reverse transcribed using QuantiFast kit (Qiagen, Hilden, Germany) after pretreatment with DNAse to eliminate any potential contamination with genomic DNA.

### Real-Time (RT)-PCR and Absolute Quantification of CD3γ and Cytokeratin 8

Real Time (RT)-PCR analysis was performed on a Rotor Gene 6000 real time rotary analyzer (Corbett Life Science) using SYBR green methodology using the following primers:

Cyto8_for 5′-GCTCCAGGCTGAGATTGAGGGCT-3′

Cyto8_rev 5′-TCCAGCTCGGACAACTTGGCG-3′

CD3γ_for 5′-CGTCCTTGCTGTTGGGGTCTACT-3′

CD3γ_rev 5′-TTGAGGGGCTGGTAGAGCTGGTC-3′

5′- CGCAGCTCAGGAAGAATGTG-3′

5′- TGAAGTACACTGGCATTGACG-3′

All values were normalized against the geometric mean of two reference genes (GAPDH and ACTB) as described previously [16]. For amplification Taq-Polymerase with an error rate of 0.22×10^−4^ was used (Fermentas/Thermo Scientific, USA). All primers were synthesized by Biomers (Germany).

### Analysis of Gene Expression Profile Using Real Time PCR Based Array

Copy numbers of mRNA for 48 different genes were analyzed using a real time PCR based customized microarray for the Corbett Rotor Gene 6000 including antimicrobial peptides (e.g. human defensins), pattern recognition receptors (e.g. Toll-like receptors), chaperones, stress genes and cytochromes. Data was analyzed using the RT^2^ Profiler PCR Array Data Analyzer 3.5 provided by SABiosciences (Maryland, USA). All analyses were performed in triplicate. CT values were used to calculate mRNA copy numbers. All analysis were normalized against ACTB, GAPDH, RPL13A and VIL1.

### Methylated DNA Immunoprecipitation (MeDIP) and qPCR Analyses

Analyses of selected genomic loci of genes found to be differentially regulated in our PCR array analyses were performed by MeDIP. Therefore genomic DNA was isolated by phenol:choroform:isoamylalcohol extraction. Subsequently 300 µl fractions of DNA (100 ng/µl) were sheared on ice by ultrasonic treatment using the Diagenode Bioruptor UCD-200 (12 cycles, 30 s “ON”, 30 s “OFF”) to obtain a fragment size between ∼200–400 bp.

After denaturation DNA was then immunoprecipitated using 1 µg of mouse monoclonal anti-5meC antibody 33D3 (Diagenode MAb-081-100). For later comparison with immunprecipitated DNA sheared “input” DNA samples were collected prior to immunoprecipitation. Subsequently immunocomplexes were separated from suspension using DiaMag protein A-coated magnetic beads (Diagenode), leading to enrichment of methylated DNA fragments. After washing and purification, samples were analysed using quantitative real time PCR (qPCR) on a Corbett Rotor Gene 6000.

To design primer pairs for amplicons, which correspond to putative sites with dynamic 5meC signatures, we screened RefSeq sequences of selected genes for the presence of CpG-rich regions 5000 bp up- and downstream of the transcription start site using EMBOSS CpGplot (http://www.ebi.ac.uk/Tools/emboss/cpgplot/index.html).

Primer pairs used are provided in Table S1. Control primers (GAPDH, TSH2B) were purchased from Diagenode (pp-1044/pp-1041).

PCR conditions were as follows: 95°C for 7 min, 5 touch-down cycles [95°C for 15s, 70–60°C for 15s (decrement of 2°C per cycle), 60°C for 30s] followed by 40 cycles of [95°C for 15s, 60°C for 30s]. Melting of PCR product was performed using a temperature gradient from 55°C to 95°C, rising in 0.5°C increments.

The recovery of 5meCpG DNA from total “input” DNA following MeDIP experiments was calculated as follows:
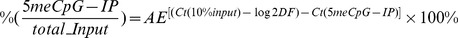



Abbreviations: AE (amplification efficiency); Ct (cycle threshold values obtained from exponential phase of the PCR reaction); the dilutional factor (DF) 10 corresponds to 10% “input” sample – thus the resulting compensatory factor in our experiments was 3.32.

### Pyrosequencing

DNA methylation signatures of promoter segments of NOD1, DNMT3A, HSPA1A, IL10, TGFB1 and EPCAM were analysed using pyrosequencing of bisulfite converted DNA. Details of pyrosequencing assays used including primer sequences and QIAGEN assay names are provided in Table S2. After standard sodium bisulphite conversion using the EZ DNA Methylation-Gold Kit (Zymo Research, USA) PCR was performed in a PTC-225, Cycler (MJ Research). Each PCR reaction contained 10 ng bisulphite treated DNA, 1x Maxima Hot Start PCR Master Mix (Thermo Scientific, USA),1x primer mix for the Qiagen assays,1 µM forward primer and 1 µM reverse primer for IL10. PCR conditions were as follows: 95°C for 15 min, followed by 45 cycles of 94°C for 30s, 56°C for 30s, 72°C for 30s, and, finally, 72°C for 10 min. PCR products were verified by agarose electrophoresis. Pyrosequencing methylation analysis was conducted using the PyroMark Q24 (Qiagen, Germany) according to the manufacturer’s protocol. The level of methylation was analysed using PyroMark Q24 2.0.6 Software (Qiagen), which calculates the methylation percentage (mC/(mC+C)) for each CpG site, allowing quantitative comparisons (mC is methylated cytosine, C is unmethylated cytosine). Non-CpG cytosine residues and a standard fully methylated DNA (Zymo Research, USA) were used as controls to verify bisulfite conversion.

### Statistical Analysis

Mean expression or methylation levels in each biopsy were obtained from duplicate real time PCR or pyrosequencing measurements. Results are presented as boxplots or barcharts displaying mean +/− standard error of mean (SEM). Calculation of significant differences between groups was performed using an unpaired *t-test* for values with Gaussian distribution. Mann-Whitney test was utilised for values without Gaussian distribution. Kolmogorov-Smirnov test was used to determine Gaussian distribution. Values for *P*<0.05 were considered statistically significant. All analysis was performed using Graphpad Prism version 5 (Graphpad Software, San Diego, CA).

## Supporting Information

Table S1 **Primer sequences used for MeDIP qPCR analysis.**
(PDF)

Table S2 **Primer sequences and assay details for pyrosequencing analysis.**
(PDF)
