# Supplementary material for: DNA Methylation Analysis in the Intestinal Epithelium—Effect of Cell Separation on Gene Expression and Methylation Profile
Source: PLoS One. 2013 Feb 8;8(2):e55636. doi: 10.1371/journal.pone.0055636 (PMC3568120; doi:10.1371/journal.pone.0055636)
Supplement: Table S1 — Primer sequences used for MeDIP qPCR analysis. (PDF) [file pone.0055636.s001.pdf]

Gene	Primer name	Primers (5'-3')
**IL10**	IL10_218for	tgcaccagggaacttgccca
	IL10_218rev	gccttggcctcccagagtgc
	IL10_50for	cgggcacagtggctcatgcc
	IL10_50rev	gccttggcctcccagagtgc
**PECAM1**	PECAM1_131for	gcccagccgttaattctattc
	PECAM1_131rev	aggaagtagggggcaaagag
	PECAM1_90for	gggagaagtgaccagagcaa
	PECAM1_90rev	catgactcgctcagcagaag
**TLR4**	TRL4_for	ttcaccaagcccaggcagaggt
	TLR4_rev	ggcgcgaggcagacatcatcc
**iNOS**	NOS_for	ggctgccagtgtgttcataa
	NOS_rev	tctgagccttctcaaagagga
**DNMT3**	DNMT3a_for	ttggccctgtagagcagagt
	DNMT3a_rev	ctgcagttctcccgacctc
	DNMT3a02_for	cctggccttatgggtaggtt
	DNMT3a02_rev	cggggaggcatacttcact
	DNMT3a03_for	ccgggtttgaaaagagtgaa
	DNMT3a03_rev	agctgaagctgcctccaac
**NOD1**	NOD1_for	cgctgggtaggcacttttac
	NOD1_rev	ggaactggctgcgactacag
**TGF-β**	TGFb1_for	aagaccacccaccttctggt
	TGFb1_rev	gaggtcctcagggagaagg
**HSPA1A**	HSPA1A_for	ccctgctcagaactctccag
	HSPA1A_rev	ccgacccttcctgtcaatta
