# Supplementary material for: DNA Methylation Analysis in the Intestinal Epithelium—Effect of Cell Separation on Gene Expression and Methylation Profile
Source: PLoS One. 2013 Feb 8;8(2):e55636. doi: 10.1371/journal.pone.0055636 (PMC3568120; doi:10.1371/journal.pone.0055636)
Supplement: Table S2 — Primer sequences and assay details for pyrosequencing analysis. (PDF) [file pone.0055636.s002.pdf]

Gene	Assay Name	Sequence to Analyse	No.of CpG	Primers (5'-3')
**NOD1**	Hs_NOD1_01_PM<br>(Qiagen)	CGCRCAGGGCCGAGTGGAC<br>AGGCCCAAGTTCG	3	included in the assay
**DNMT3A**	Hs_DNMT3A_01_PM<br>(Qiagen)	CGCGCCGCAACCCCCAGCCA<br>GGTGCCGCCGCGG	6	included in the assay
	Hs_DNMT3A_02_PM<br>(Qiagen)	CGGGGGCGGGGAGGCGGC<br>AGGGCCGGTGGGAGGATCT<br>CCG	5	included in the assay
	Hs_DNMT3A_03_PM<br>(Qiagen)	TTACCACTGCCCGGGCTCCC<br>GGCCGGCTGCTCTTCCTGTC<br>CCCCGAGGGCG	5	included in the assay
**HSPA1A**	Hs_HSPA1A_01_PM<br>(Qiagen)	CGATGCTGACCGCCTGCGCT<br>TCAGCG	4	included in the assay
	Hs_HSPA1A_02_PM<br>(Qiagen)	TCGTCACGGAGACCCGCCTT<br>TTCCCTTCTGAGCCAATCAC<br>CGAGCTCG	5	included in the assay
**TGFB1**	Hs_TGFB1_01_PM<br>(Qiagen)	TCAGTATCCCACGGAAATAA<br>CCTAGATGGGCGCG	3	included in the assay
**EPCAM**	Hs_EPCAM_03_PM<br>(Qiagen)	GCAGGGAACGGAGTGGCCA<br>CGTCCAGGTTTCCTGCGGCC<br>ACCGAACCGG	5	included in the assay
**IL10**	IL 10-145	CGGGAAACCTTGATTGT	1	fwd: AGAAGGAGGAGTTTTAAGGAGAAA<br>rvs: (Biotin)TCATTCATTAAAAAACCACAATCA<br>seq: AGGAGAAAAAATTTTGTGT
	IL 10-355	CGCACAGAACAGCTGTT	1	fwd: GAATACGCGAATGAGAATTTATAG<br>rvs: (Biotin)TTTCCTAAAAAAACAACTATTCTA<br>seq: TTTATAGTTGAGGGTTTTTG
	IL 10-387	CGCGAATGASAACCCACAGC<br>TGAGGGCCTCTGCGC	3	fwd: TGTATTTTGGAATGGGTAATTTG<br>rvs: (Biotin)AAAACCCTCAACTATAAATTCTCA<br>seq: TATTGTGATTTAGGAATA
	IL 10-408	ACGTCACTGTGACCTAGGAA<br>CACGCGAA	3	fwd: TGTATTTTGGAATGGGTAATTTG<br>rvs: (Biotin)AAAACCCTCAACTATAAATTCTCA<br>seq: GGAATGGGTAATTTGTTT
